# Signaling Modulation by *miRNA-221-3p* During Tooth Morphogenesis in Mice

**DOI:** 10.3389/fcell.2021.697243

**Published:** 2021-08-25

**Authors:** Yam Prasad Aryal, Tae-Young Kim, Eui-Seon Lee, Chang-Hyeon An, Ji-Youn Kim, Hitoshi Yamamoto, Sanggyu Lee, Youngkyun Lee, Wern-Joo Sohn, Sanjiv Neupane, Jae-Young Kim

**Affiliations:** ^1^Department of Biochemistry, School of Dentistry, Kyungpook National University, Daegu, South Korea; ^2^Department of Oral and Maxillofacial Radiology, School of Dentistry, Kyungpook National University, Daegu, South Korea; ^3^Department of Dental Hygiene, College of Health Science, Gachon University, Incheon, South Korea; ^4^Department of Histology and Developmental Biology, Tokyo Dental College, Tokyo, Japan; ^5^School of Life Sciences, BK21 Plus KNU Creative BioResearch Group, Kyungpook National University, Daegu, South Korea; ^6^Pre-Major of Cosmetics and Pharmaceutics, Daegu Haany University, Gyeongsan-si, South Korea; ^7^Department of Biochemistry and Cell Biology, Stony Brook University, Stony Brook, NY, United States

**Keywords:** miRNA, tooth development stage, signaling modulation, cellular events, tooth morphogenesis

## Abstract

miRNAs are conserved short non-coding RNAs that play a role in the modulation of various biological pathways during tissue and organ morphogenesis. In this study, the function of *miRNA-221-3p* in tooth development, through its loss or gain in function was evaluated. A variety of techniques were utilized to evaluate detailed functional roles of *miRNA-221-3p* during odontogenesis, including *in vitro* tooth cultivation, renal capsule transplantation, *in situ* hybridization, real-time PCR, and immunohistochemistry. Two-day *in vitro* tooth cultivation at E13 identified altered cellular events, including cellular proliferation, apoptosis, adhesion, and cytoskeletal arrangement, with the loss and gain of *miRNA-221-3p*. qPCR analysis revealed alterations in gene expression of tooth-related signaling molecules, including β*-catenin*, *Bmp2*, *Bmp4*, *Fgf4*, *Ptch1*, and *Shh*, when inhibited with *miRNA-221-3p* and mimic. Also, the inhibition of *miRNA-221-3p* demonstrated increased mesenchymal localizations of pSMAD1/5/8, alongside decreased expression patterns of *Shh* and *Fgf4* within inner enamel epithelium (IEE) in E13 + 2 days *in vitro* cultivated teeth. Moreover, 1-week renal transplantation of *in vitro* cultivated teeth had smaller tooth size with reduced enamel and dentin matrices, along with increased cellular proliferation and *Shh* expression along the Hertwig epithelial root sheath (HERS), within the inhibitor group. Similarly, in 3-week renal calcified teeth, the overexpression of *miRNA-221-3p* did not affect tooth phenotype, while the loss of function resulted in long and slender teeth with short mesiodistal length. This study provides evidence that a suitable level of *miRNA-221-3p* is required for the modulation of major signaling pathways, including Wnt, Bmp, and *Shh*, during tooth morphogenesis.

## Introduction

The well-defined stages in which tooth development progresses require an intricate and reciprocal signaling regulation between both the dental epithelium and neural-crest-derived mesenchyme (Balic and Thesleff, 2015). This reciprocal signaling frequently involves signaling via Bmp, *Shh*, Wnt, and Fgfs throughout the various stages of tooth development including initiation, bud, bell, cap, and root morphogenesis (Jussila and Thesleff, 2012). In particular during bud to cap transition Bmp, *Shh*, Wnt, and Fgf signaling play a key role in maintaining the tooth crown structure through enamel knot (EK) morphogenesis (Balic and Thesleff, 2015). In addition, the very same signaling governs hard tissue formation via regulation of differentiation and secretion stages, and tooth root morphogenesis by maintenance and proliferation of HERS, which occurs at the cervical loop of the enamel organ (Lungová et al., 2011; Li et al., 2017). In particular, Wnt signaling is associated with the determination of tooth number, shape, and hard tissue formation; whereas cellular behavior, such as proliferation, apoptosis, differentiation, and migration of cells, is regulated by Fgf, *Shh*, and Bmp signaling (Liu et al., 2008; Balic and Thesleff, 2015). Disruption of the precise signaling pathways during tooth development will lead to abnormal tooth crown and root morphogenesis (Liu and Millar, 2010; Wang et al., 2012; Wang and Feng, 2017). Therefore, the use of activators and inhibitors on the signaling regulations, such as overexpression vectors or siRNAs, are being investigated in a range of model organs, including the tooth which may provide crucial knowledge related to tooth crown and root morphogenesis (Aryal et al., 2020; Neupane et al., 2020). To date, the signaling involved that modulates the tooth crown and root has yet to be broadened to specific molecules, such as miRNA. Consequently, functional studies including miRNA, siRNA, lncRNA, exosome, and so on have been utilized to define the developmental processes in tooth organogenesis (Jiang et al., 2017; Aryal et al., 2020; Huang et al., 2020) and therefore, require further research.

miRNAs are conserved short non-coding RNAs, an average of 22 nucleotides in length. Mature miRNA are up to several hundred nucleotides in length, called pri-miRNA, before being processed by the enzymes Drosha and Dicer (Ha and Kim, 2014; Wang et al., 2016). The biogenesis of miRNA is a complex and dynamic process, and any disruption to these processes could result in impaired production of mature miRNA/this, in turn, can lead to an imbalance in tissue homeostasis and disease progression (Trionfini et al., 2015; Barwari et al., 2016; Wang et al., 2016). The role of miRNAs in the modulation of various biological pathways including tissue and organ morphogenesis, have been extensively studied (Bartel, 2004; Andl et al., 2006; Yi et al., 2006; Cao et al., 2010; Wang et al., 2016; Rahmanian et al., 2019; Barbu et al., 2020). The involvement of miRNAs during tooth morphogenesis, which involves complex and reciprocal interactions between epithelial and mesenchymal tissue (Jussila and Thesleff, 2012; Huang et al., 2020), has also been identified. During mammalian tooth development, miRNAs regulate dental epithelial and mesenchymal cell differentiation during the early and secretory stages of odontogenesis (Cao et al., 2010; Li et al., 2015; Neupane et al., 2020). Functional studies conducted on different miRNAs highlight their roles during tooth morphogenesis. For example, *miR-135a* regulates Bmp signaling (Kim et al., 2014), *miR-31*, and *miR-138* regulate differentiation of adult stem cell niche in mice incisors (Jheon et al., 2011), and *miR-31, miR-140, MiR-141, miR-143, miR-145, miR-455, miR-689, miR-711, miR-720*, and *miR-875* regulate ameloblast and odontoblast differentiation (Michon et al., 2010; Liu et al., 2013, 2014; Funada et al., 2020). More than 700 miRNAs have been identified to date (Rahmanian et al., 2019), and it is pertinent to identify the function of each of these miRNAs to understand their precise roles within the signaling pathways during tooth development. In this study, we selected *miRNA-221-3p* as one of the candidate miRNA due to its high expression identified by miRNA-Microarray experiment utilizing the embryonic mice molar at E14 (unpublished data). This prompted the investigation into the role of *miRNA-221-3p* in signaling modulation during tooth development, which is important for understanding the regulatory pathways involved in tooth development.

In this study, *miRNA-221-3p*, one of the conserved miRNA, was investigated via an *in vitro* organ cultivation system, and a loss and gain of function assay using *miRNA-221-3p* specific inhibitors and its mimics. *miRNA-221-3p* is has been well studied in cancer (Liu et al., 2014; Tao et al., 2014; Zheng et al., 2014; Ihle et al., 2015; Wang et al., 2019), wound healing (Xu et al., 2020), and bone metabolism (Maeda et al., 2017), through modulation of signaling pathways. More importantly, *miRNA-221-3p* is predicted to regulate Shh and Wnt signaling through *Ptch1* and *Dkk2* inhibition, respectively (Agarwal et al., 2015). In cancer cells, miRNA also regulates proliferation and apoptosis (Ihle et al., 2015; Yang et al., 2019), and these cellular events are also important for tooth morphogenesis. Given the important role of Wnt and Shh signaling in tooth development (Jussila and Thesleff, 2012; Balic and Thesleff, 2015), it is pertinent to investigate signaling modulation of *miRNA-221-3p* in tooth development. Therefore, in this study, we have elucidated the functional roles of *miRNA-221-3p* during tooth crown and root morphogenesis.

## Materials and Methods

### Animals

All experiments involving animals were performed according to the guidelines of the Kyungpook National University, School of Dentistry, Intramural Animal Use and Care Committee (KNU-2020-0107). Mice were kept in an optimal environment, maintaining relative humidity (55%) and temperature (25°C) with access to food and water *ad libitum*. For time-mated pregnant mice, embryonic day 0 (E0) was assigned to the day in which a vaginal plug was confirmed. All experiments described in this study were independently performed in triplicate as a minimum.

### *In situ* Hybridization

mRNA and miRNA *in situ* hybridizations were performed as described previously (Neupane et al., 2020). For miRNA *in situ* hybridizations, a 5′- and 3′- DIG-labeled miRCURY LNATM miRNA custom detection probe was hybridized using the miRNA ISH buffer set according to the manufacturer’s instructions (Exiqon, Skelstedet, Denmark, cat no. 90000). The miRCURY LNATM miRNA custom detection probe for 221-3P (/5DigN/AAACCCAGCAGACAATGTAGCT/3Dig_N/) was purchased from Qiagen (Qiagen, Hilden, Germany, cat. no. 339115 YCD002344-BCG). For section *in situ* hybridizations, digoxigenin (DIG)-labeled antisense RNA probes were hybridized overnight to examine the detailed expression patterns of genes as described previously (Aryal et al., 2020; Neupane et al., 2020).

### *In vitro* Organ Cultivation and Renal Capsule Transplantation

E13 embryos that are at the bud stage of tooth development, underwent microdissection isolation of the embryonic tooth germs. These were subsequently cultured in DMEM (HyClone, Logan, UT, United States; cat. no.-SH30243.01) with 10% fetal bovine serum (Hyclone, Logan, UT, United States) and antibiotics, and cultivated using a modified Trowell’s culture method for 2 days, as previously described (Aryal et al., 2020; Neupane et al., 2020). To check the loss and gain of function of *miRNA-221-3p*, tooth germs were cocultivated with transfecting 400 nM of the inhibitor (Qiagen, Hilden, Germany, miCURY LNATM miRNA Power Inhibitor, cat no. 339131 YI04102471-DDB) or (mimic miCURY LNATM miRNA Mimic, cat no. 339173 YM00471077-ADB) of the *miRNA-221-3p* as previously described (Neupane et al., 2020). After transfection of miRNA at E13 for 2 days, the cultivated tooth germs were transplanted into the kidney capsule of adult ICR male mice as previously described (Aryal et al., 2020; Neupane et al., 2020). Differing groups of mice were sacrificed at either 1-week or 3-week post-transfection, and the calcified teeth were harvested.

### Histology and Immunohistochemistry

Histology and immunostaining were carried out as described previously (Aryal et al., 2020; Neupane et al., 2020). Hematoxylin and eosin (H&E) staining were employed to examine the detailed morphology of the *in vivo* and *in vitro* tooth. Primary antibodies were directed against Ki67 (Cat# RM-9106-s, Thermo Scientific, United States); ROCK1 (Cat# ab45171, Abcam); E-cadherin (Cat# AF748, R&D Systems, United States); β-catenin (Cat# 8814S, Cell Signaling Technology, MA, United States); pSMAD (Cat# 9511S, Cell Signaling Technology, MA, United States); AMELX (Cat# ab153915, Abcam, United States) and NESTIN (Cat# ab11306, Abcam, United States). The secondary antibodies used in this study were biotinylated goat anti-rabbit or anti-mouse IgG. Immunocomplexes were visualized using a diaminobenzidine tetrahydrochloride (DAB) reagent kit (Cat# 00-2014; Zymed Laboratories, United States) and goat Anti-Rabbit Alexa Fluor@488 (Cat# ab150077, Abcam, United States); goat Anti-Mouse Alexa Fluor@647 (Cat# ab150115, Abcam, United States). The DAB stained slides were mounted with xylene based mounting medium Malinol and immunofluorescent slides were mounted with VECTASHIELD^®^ Antifade Mounting Medium containing DAPI (Cat# H-1200-10, Vector laboratories) and cover slipped.

Histological sections were photographed using a Leica DM2500 with and without fluorescent setting. ImageJ^1^ was used to measure the fluorescence of the images as described previously (Neupane et al., 2021). Briefly, fluorescence images were split for different channels and integrated density and background fluorescent were measured in defined region of interests. The total corrected cell fluorescence was measured as: [Integrated density-(area X background fluorescence)]. Fluorescence was measured in sections obtained from minimum of three independent experiments (*n* = 3) for each group. Data were evaluated for significance using unpaired, two-tailed *t*-test. A *p-*value of <0.05 indicates significance.

For colocalization analysis the correlation index (Pearson’s correlation coefficient) of colocalized pixels between the green (β-Catenin) and blue (DAPI for nucleus) colors were measured in ImageJ and plotted as graph in GraphPad Prism as described previously (Neupane et al., 2021). Briefly, the color of image was split into component channels and the green and blue channel were used to evaluate the colocalization using ImageJ plugin “Colocalization.” If the green (β-Catenin) was localized in the nucleus, it will colocalize with the blue (DAPI for nucleus) that will produce the correlation index of colocalization. Higher the correlation index, higher will be the colocalization and vice versa.

The number of Ki67 positive cells in DAB stained sections were counted in defined area of 200 × 200 μm^2^ in mesenchyme adjacent to the enamel knot. Whereas number of Ki67 positive cells from fluorescent labeled sections were presented as % of Ki67 positive cells (green) in total numbers of nuclei (blue) in the given field. GraphPad Prism (version 8) was used to calculate the test statistics and plot the graph. Briefly, after splitting the channels to blue (DAPI for nuclei) and green (Ki67) in ImageJ, the individual channels were processed through thresholding, binary processing (close, open, and watershed) and finally particle measurement (Supplementary Figure 1). This will give an approximate number of DAPI positive cells from blue channel and Ki67 + cells from the green channel which was used to calculate the % Ki67 positive cells in total number of cell.

### Quantitative RT-PCR (qPCR)

Quantitative RT-PCR (qPCR) was performed as previously described (Aryal et al., 2020; Neupane et al., 2020). RNA was extracted from E13 + 36 and 48 h incubation with *miRNA-221-3p* inhibited and mimic transfected tooth germs, and qPCR was subsequently performed to examine the loss and gain of function effect of *miRNA-221-3p*. The mRNA and the miRNA qPCR results for each sample were normalized to those of *Hprt* and *snoRNA135*, respectively. Data were represented as means ± SD. Mean expression levels were determined by comparing experimental and control groups using the Student’s *t*-test. A *p-*value of <0.05 indicates significance. The experiments are repeated at least three times to check the reproducibility of the data. Primer sequences used in the qPCR assays are detailed in Supplementary Table 1.

## Results

### *miRNA-221-3p* Modulates Cellular Events During Tooth Development

**FIGURE 1:**
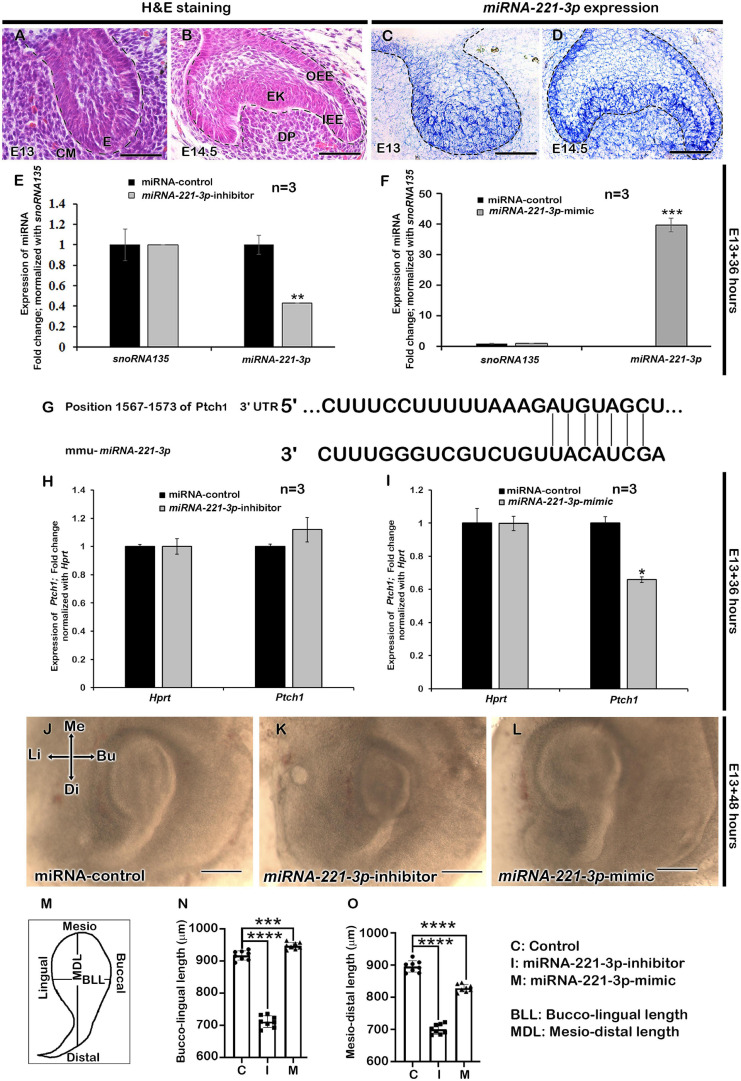
Expression of *miRNA-221-3p* and loss and gain of function in developing tooth germ. H&E staining showing the developing tooth germ at the bud and cap stage **(A,B)**. Section *in situ* hybridization showing expression of *miRNA-221-3p* along the invaginated epithelium at the bud stage **(C)** and along the IEE, OEE, EK, DP, and cervical loop at the cap stage **(D)**. Mimic and inhibitor significantly altered the miRNA expression level of *miRNA-221-3p* at E13 for 36 h (**E,F**, *n* = 3) (corresponding to data in Supplementary Table 2A). Conserved seed sequence of *miRNA-221-3p* with *Ptch1* gene **(G)**. The expression level of *Ptch1* increased with *miRNA-221-3p* inhibition (*n* = 3), while decreased with mimic (**H,I**, *n* = 3) (corresponding to data in Supplementary Table 2B). The culture of tooth germs with mimic and inhibitor at E13 for 2 days show alteration in the length of developing tooth mesio-distally and bucco-lingually (**J–O**, *n* = 8). The dotted lines define the boundary of epithelium and mesenchyme **(A–D)**. H&E, hematoxylin and eosin staining; E, enamel organ; IEE, inner enamel epithelium; OEE, outer enamel epithelium; EK, enamel knot; DP, dental papilla; Me, mesial; Di, distal; Bu, buccal; Li, lingual. Data were evaluated for statistical significance using unpaired, two-tailed *t*-test. *, **, ***, and **** indicate *p* < 0.05, 0.01, 0.001, and 0.0001, respectively. Scale bars: 500 μm **(J–L)**, 50 μm **(A–D)**.

**FIGURE 2:**
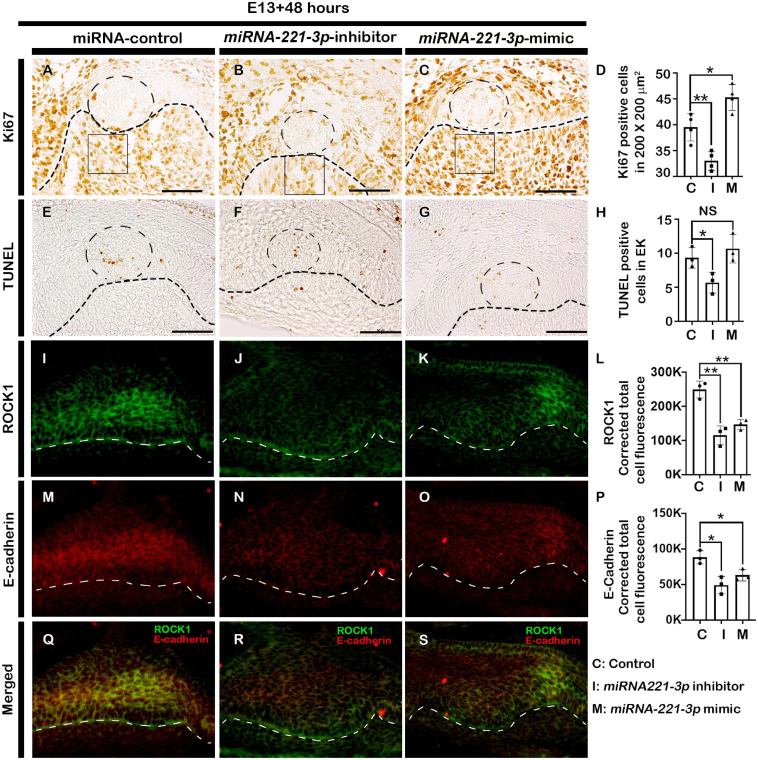
Altered cellular events after loss and gain of function of *miRNA-221-3p*. The cellular proliferations especially at the EK and dental mesenchyme adjacent to the IEE increased in the mimic and decreased in the inhibitor-treated tooth germs (**A–D**, *n* = 4). Loss and gain of *miRNA-221-3p* at E13 for 2 days show decreased apoptotic cells along the EK of inhibitor-group compared to control and mimic (**E–H**, *n* = 3). Localizations of ROCK1 (green) **(I–L)** (*n* = 3) and E-cadherin (red) (**M–O**, *n* = 3) along the enamel organ, showing decreased intensities in the inhibitor and mimic specimens, compared to control (**Q–S**, *n* = 3). The dotted circles in the IEE **(A–C,E–G)** define the EK, the dotted lines define the boundary of epithelium and mesenchyme **(A–O)** and the squares **(A–C)** defines the area where Ki67 positive cells are counted. EK, enamel knot. Data were evaluated for statistical significance using unpaired, two-tailed *t*-test. * and ** indicate *p* < 0.05 and 0.01, respectively, NS, not significant. Scale bars: 50 μm **(A–O)**.

The developing tooth germ is at the bud and cap stage at the embryonic stage E13 and E14.5, respectively (Figures 1A,B). During bud stage, the expression of *miRNA-221-3p* was restricted along the invaginated epithelium (Figure 1C) with intense expression noted along the enamel knot (EK), IEE, outer enamel epithelium (OEE), and dental papilla (DP) at cap stage (Figure 1D). These specific expression patterns suggest a role for *miRNA- 221-3p* in the bud to cap stage transition of tooth development that ultimately regulates the signaling of the tooth crown and root morphogenesis. To evaluate the functional role of *miRNA-221-3p* in tooth development, the *in vitro* cultivation of tooth germ with *miRNA-221-3p* inhibitor and its mimic was performed. This significantly altered the expression level of miRNA at E13 for 36 h (Figures 1E,F). According to the database TargetScan, *miRNA-221-3p* is broadly conserved among vertebrates and predicted to target *Ptch1* (Figure 1G and Supplementary Figure 2; Agarwal et al., 2015). Inhibition and mimic of *miRNA-221-3p* increased and decreased the level of *Ptch1*, which is in agreement with the seed sequence prediction of Agarwal et al. (2015) (Figures 1G–I). A 2-day cultivation of tooth with an inhibitor and mimic resulted in a decrease and increase in tooth size, respectively, along the buccolingual and mesiodistal axis, in comparison to the control (Figures 1J–O). This altered tooth size during *in vitro* organ cultivation, suggests a possible change in cellular behaviors, including cell proliferation, apoptosis, and cell migration, which are all crucial for determining tooth size (Setkova et al., 2006). Therefore, to understand the precise role of *miRNA-221-3p* in the cellular events including proliferation, apoptosis, and cell adhesion, we utilized Ki67, E-cadherin, and ROCK1 immunohistochemistry and TUNEL assay. Compared to the control (Figure 2A), the cellular proliferation, in particular at the IEE and dental mesenchyme adjacent to the IEE, was decreased in the inhibitor-treated specimens (Figures 2B,D and Supplementary Figure 3). However, an increase in the mimic-treated specimens was observed (Figures 2C,D). Similarly, the TUNEL positive cells are significantly decreased in the inhibitor-treated specimens compared to control and mimic (Figures 2E–G and Supplementary Figure 3). Meanwhile, the number of apoptotic cells in the EK was similar to that in the control and mimic-treated specimens (Figures 2E,G,H). To understand these cellular events further, we evaluated the localization patterns of E-cadherin and ROCK1 (Figures 2I–L). ROCK1, which is a downstream target of RhoA small GTP-binding proteins, regulates cell adhesion, cytokinesis, cell proliferation, and apoptosis and regulates actin cytoskeleton dynamics forming a complex with E-cadherin (Zohrabian et al., 2009; Smith et al., 2012). In addition, ROCK1 and E-cadherin primarily localize in the enamel organ and regulate the cellular proliferation in the IEE (Aryal et al., 2020). In our study, the colocalization of ROCK1 and E-cadherin was evenly distributed along the IEE in the control specimen (Figures 2I,M,Q). In contrast, inhibiting and mimicking of *miRNA-221-3p* decreased the intensity of fluorescence of E-cadherin and ROCK1 in the IEE (Figures 2J–L,N–P,R,S). These observations suggest that *miRNA-221-3p* modulates proliferation and determines the size of the developing tooth through ROCK1 and E-cadherin mediated cell adhesion. This will eventually regulate the tooth crown and root morphogenesis in later stages of tooth development.

### miRNA-221-3p Modulates Tooth Development-Related Signaling Molecules

**FIGURE 3:**
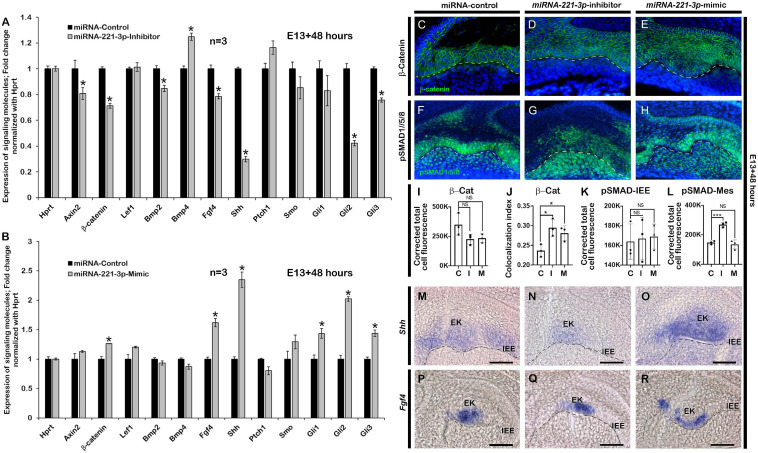
Altered expressions of signaling molecules after loss and gain of function of *miRNA-221-3p*. Signaling molecules including *Axin2*, β*-catenin*, *Bmp2*, *Bmp4*, *Fgf4*, *Shh, Gli1, Gli2*, and *Gli3* are significantly altered in the inhibitor and mimic-treated specimens (**A,B**, *n* = 3) (corresponding to data in Supplementary Table 2B). Epithelial localization of β-catenin (green) along IEE, showing comparatively increased nuclear localization of β-catenin in the inhibitor and mimic-treated specimens compared to control (**C–E**,**I,J**, *n* = 3). DAPI (blue) stains the nuclei. Epithelial and mesenchymal localization pattern of pSMAD1/5/8 (green) after inhibition and mimic of *miRNA-221-3p*, showing intense mesenchymal localization in the inhibitor-treated specimens compared to control and mimic (**F–H,K,L**, *n* = 3). Section *in situ* hybridization of *in vitro* cultivated tooth germs showing expressions of *Shh* (**M–O**, *n* = 3) and *Fgf4* (**P–R**, *n* = 3) along the EK and IEE. β-Cat, β-Catenin; EK, enamel knot; IEE, inner enamel epithelium; Mes, mesenchyme. Dotted line demarcate the boundary of epithelium and mesenchyme **(C–H,M–R)**. Data were evaluated for statistical significance using unpaired, two-tailed *t*-test. * indicate *p* < 0.05; NS, not significant. Scale bars: 50 μ **(C–N)**.

There is high conservation of signaling molecules belonging to Bmp, Fgf, Shh, and Wnt pathways to achieve proper tooth development from initiation to eruption (Jussila and Thesleff, 2012). To evaluate whether the inhibition and mimicking of *miRNA-221-3p* during odontogenesis altered tooth development-related signaling molecules, we employed qPCR at E13 + 2 days to evaluate the expressions of these signaling molecules as described previously (Aryal et al., 2020; Neupane et al., 2020). Tooth germ culture at E13 + 2 days with inhibitor upregulated the *Ptch1* and with the mimic downregulated the *Ptch1* expression consistent with our hypothesis that *miRNA-221* targets *Ptch1*. Meanwhile *Axin2*, β*-catenin*, *Bmp2*, *Fgf4*, *Shh*, *Smo*, *Gli1*, *Gli2*, and *Gli3* were downregulated, whereas *Bmp4* was upregulated in the inhibitor-treated specimen (Figure 3A). In contrast, β*-catenin*, Lef1, *Fgf4*, *Shh*, *Smo*, *Gli1*, *Gli2*, and *Gli3* were upregulated, with *Bmp4* downregulated in the mimic-treated specimen (Figure 3B). Except *Shh* target genes, the expressions of Wnt, Bmp, and Fgf-related signaling molecules were almost similar in both 36 and 48 h in *in vitro* cultivated tooth germs (Figures 3A,B and Supplementary Figure 4). Wnt/β-catenin signaling negatively regulates the odontogenic epithelial cell proliferation during the bell stage of tooth development (Lu et al., 2019) and has a role in crown and tooth root morphogenesis. To determine the role of *miRNA-221-3p* in Wnt signaling modulation, immunohistochemistry with β-catenin was performed in E13 + 2 days *in vitro* cultivated tooth germs. The active form of β-catenin was localized in the cytoplasm and nucleus of IEE in the control tooth at E13 + 2 days (Figures 3C,I,J). However, an increase in the localization of β-catenin was observed comparatively within the nucleus in the inhibitor and mimic-treated specimen (Figures 3D,E,I,J and Supplementary Figure 5) suggesting that Wnt signaling is negatively regulated by *miRNA-221-3p* during tooth cap and bell stages. Previous study suggested that Wnts and *Bmp4* are the key mediators of odontogenic epithelial–mesenchymal interactions, and that Wnt signaling is upstream of Fgf, Shh, and Bmp signaling during tooth development (O’Connell et al., 2012). This suggests that Bmp signaling would be indirectly affected when the levels of *miRNA-221-3p* are altered. Therefore, immunohistochemistry of pSMAD1/5/8 was performed on inhibitor and mimic to *miRNA-221-3p*-treated teeth. The epithelial localization of pSMAD1/5/8 was almost similar in all specimens, however, the mesenchymal localizations of pSMAD1/5/8 were stronger in the inhibitor-treated specimen compared to control and mimic-treated specimens (Figures 3F–H,K,L). This suggests a role for *miRNA-221-3p* during the cap and bell stages of tooth development that also affects the tooth crown and root morphogenesis. Similarly, section *in situ* hybridization was performed to examine the Shh and Fgf signaling after inhibition and mimicking of *miRNA-221-3p*. Shh activity in the developing tooth is very dynamic throughout tooth development, including tooth crown and root morphogenesis (Seppala et al., 2017). The expression of *Shh* was expanded along the entire IEE of the control specimen (Figure 3M), however, the inhibitor-treated tooth germ had confined expression patterns along the EK only (Figure 3N). However, the expression of *Shh* in the mimic-treated specimen was expanded along the EK, although its expression pattern was not continuous along the entire IEE and ectopically expressed in the dental papilla (Figure 3O), suggesting *miRNA-221-3p* modulates *Shh* in the developing tooth potentially through *Ptch1* inhibition. Meanwhile, *Fgf4*, which determines invagination of dental epithelium, tooth shape, and cusps formation, and also ameloblast and odontoblast differentiation (Li et al., 2014) was expressed along the EK of the *in vitro* cultivated tooth germs. However, the expression pattern of *Fgf4* is extended in the EK of mimic-treated tooth germ compared to the control and inhibitor (Figures 3P–R), suggesting the potential role of *miRNA-221-3p* in crown morphogenesis that ultimately affecting the root morphogenesis.

### Loss of *miRNA-221-3p* Alters Tooth Crown and Root Morphogenesis

**FIGURE 4:**
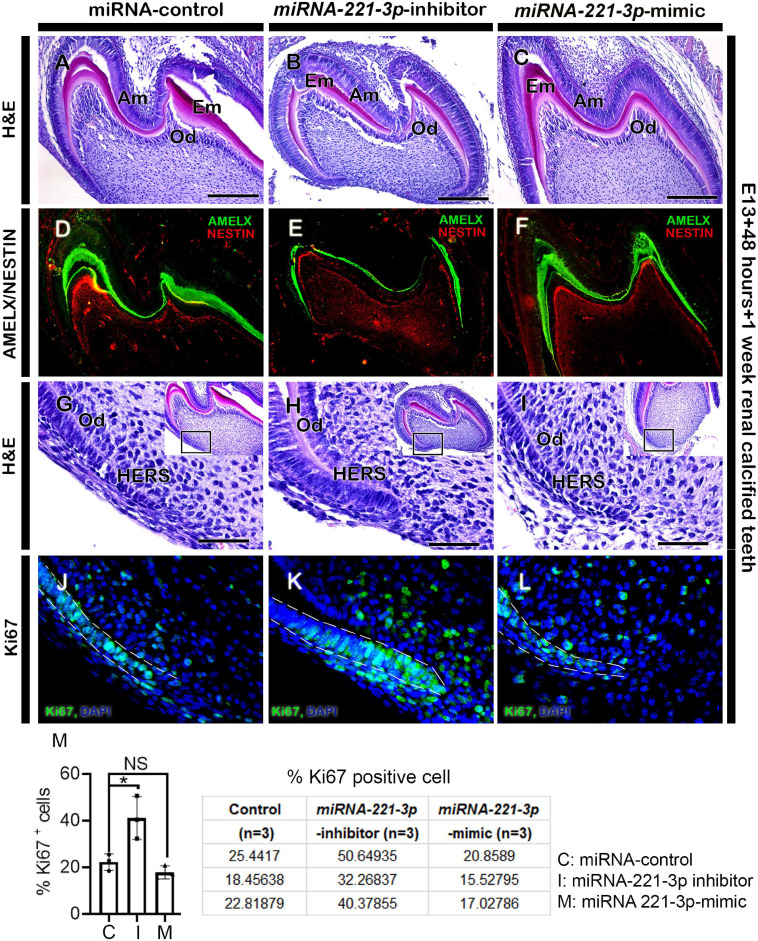
Altered histogenesis and cellular events in 1-week renal calcified teeth. H&E staining showing 1 week renal calcified teeth after loss and gain of function of *miRNA-221-3p* (**A–C**, *n* = 3). The inhibitor-treated tooth **(B)** is comparatively smaller with reduced extracellular matrix secretion compared to control and mimic **(A,C)**. The localizations of AMELX and NESTIN are weaker in inhibitor-treated specimen compared to mimic and control (**D–F**, *n* = 3). H&E staining showing HERS in 1-week calcified teeth **(G–I)**. The cellular proliferation (green) along the HERS is higher in the inhibitor-treated specimen compared to mimic and control (**J–M**, *n* = 3). DAPI (blue) stains the nuclei. Boxes in **G–I** are magnified views. Dotted lines **(J–L)** indicate the HERS. H&E, hematoxylin and eosin; Am, ameloblasts; Od, odontoblasts; AMELX, amelogenin; HERS, hertwig’s epithelial root sheath. Data were evaluated for statistical significance using unpaired, two-tailed *t*-test. ^∗^Indicates *p* < 0.05; NS, not significant. Scale bars: 200 μm **(A–F)**, 50 μm **(G–L)**.

**FIGURE 5:**
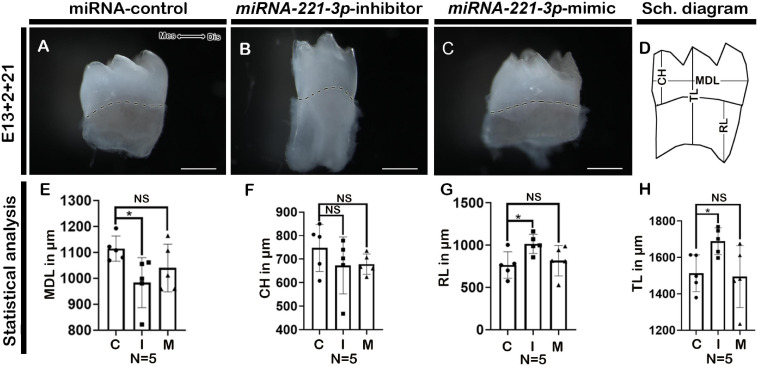
Altered tooth morphology in 3-week renal calcified teeth. Three weeks renal calcified teeth showing buccal view **(A–C)**. Schematic diagram showing MDL, CH, RL, and TL of renal calcified teeth **(D)**. Statistical analysis showing MDL, CH, RL, and TL of 3-week renal calcified tooth (**E–H**, *n* = 5). Inhibition of *miRNA-221-3p* makes tooth longer and slenderer compared to control and mimic **(A–C)**. The mesio-distal length is significantly shorter, while root length is significantly longer in the inhibitor-treated specimen **(B,E,G)**. The dotted lines demarcate the boundary of crown and root **(A–C).** MDL, mesio-distal length; CH, crown height; RL, root length; TL, total length; Mes, mesial; Dis, distal. Data were evaluated for statistical significance using unpaired, two-tailed *t*-test. ^∗^Indicates *p* < 0.05; NS, not significant. Scale bars: 500 μm **(A–C)**.

Renal capsule transplantation was utilized to determine whether inhibition and mimicking of *miRNA-221-3p* occurred during tooth crown and root development, a technique previously used (Aryal et al., 2020; Neupane et al., 2020). The developmental timeline of *in vitro* (E13 + 2 days) and 1-week renal calcified teeth was similar to the *in vivo* post-natal 3 (PN3) mice. The double-layered HERS grew apically and meets OEE and IEE leaving no interposing layers of stellate reticulum (SR) and stratum intermedium (SI) (Figures 4A–C). The ameloblasts (Am) and odontoblasts (Od) are in their secretory phase and secrete extracellular matrix (Em) (Figures 4A–C). Consistently, the inhibition of *miRNA-221-3p* demonstrated a smaller tooth with a reduction in Em secretion, while mimicking the miRNA did not show an alteration in tooth size (Figures 4A–C). Therefore, we evaluated the localization patterns of major extracellular matrix protein of enamel: amelogenin (AMELX) and odontoblast differentiation marker: NESTIN. The localization patterns of AMELX and NESTIN were almost similar in both control and mimic-treated specimens, whereas the localization intensity decreased in the inhibitor-treated specimen (Figures 4D–F). Meanwhile, the localization of NESTIN had an increase in intensity in the dental pulp cells along with secretory odontoblasts in the inhibitor-treated specimen, in comparison to the control and mimic specimen (Figures 4D–F). In addition, the epithelial cells of HERS in 1-week renal calcified teeth were columnar shaped in the inhibitor-treated specimen in comparison to control and mimic, which were cuboidal in shape (Figures 4G–I). This led us to examine the cellular proliferations along the HERS, and it was identified that cellular proliferation of HERS was comparatively higher in the inhibitor-treated specimen compared to mimic and control (Figures 4J–M and Supplementary Figure 1). As HERS play an important role in tooth root formation, we further examined the expression of the signaling molecule *Shh*, which plays an important role in HERS during tooth root formation (Nakatomi et al., 2006; Ota, 2008; Li et al., 2017). The section *in situ* hybridization demonstrated intense expression of *Shh* along the HERS within the inhibitor group compared to control and mimic (Supplementary Figure 6). To confirm whether altered proliferation and signaling in HERS in the inhibitor-treated specimen would have impacted tooth morphology, we employed 3-week renal capsule calcified teeth, and their morphometric measurements were taken (Figure 5). The particular measurements taken were crown length, mesiodistal length, root length, and absolute tooth length. Inhibition of *miRNA-221-3p* results in a longer and slenderer tooth compared to control and mimic (Figures 5A–H). However, in the inhibitor-treated specimen the mesiodistal length is significantly shorter, while the root and absolute tooth length is significantly longer (Figures 5B,E,G).

## Discussion

The complex and dynamic molecular interactions involved in tooth development are well understood and have highlighted the importance of spatiotemporal functions of signaling molecules during odontogenesis (Jussila and Thesleff, 2012; Huang et al., 2020). Experimental approaches, such as *in vitro* cultivation and loss and gain of function of signaling molecules, demonstrate their precise roles during tissue and organ morphogenesis (Jiang et al., 2017; Aryal et al., 2020; Huang et al., 2020). In this study, *miRNA-221-3p*, a conserved miRNA within the animal kingdom (Agarwal et al., 2015; Mari et al., 2016; Abak et al., 2018), was evaluated through knockdown and mimicking of function, during mice molar development. The precise expression of *miRNA-221-3p* has been identified to overlap with the expression of known transcription and paracrine factors, such as *Bmp2*, *Bmp4*, *Bmp7*, *Wnt3*, *Wnt10a*, *Wnt10b*, *Fgf4*, and *Shh* (Jussila and Thesleff, 2012; Balic and Thesleff, 2015), during important stages of tooth development (Figure 1). This demonstrates its modulating role during odontogenesis that prompted us to examine its role during molar development in detail.

The epithelial–mesenchymal interactions up to, and beyond the bud stage plays a significant role in normal tooth progression and development (Jussila and Thesleff, 2012; Huang et al., 2020). The altered signaling molecules that arise as a result of inhibition and mimicking of *miRNA-221-3p* during the bud stage of tooth development, highlighted its role in signal modulation during odontogenesis. Normally, the activation of Wnt/β-catenin signaling occurs in the dental epithelial and adjacent mesenchymal cells. However, upon inhibition, it resulted in the developmental arrest at the bud stage and short root phenotype (Liu et al., 2008; Liu and Millar, 2010; Han et al., 2011). In our study, the inhibition of *miRNA-221-3p* during bud stage showed downregulation of β*-catenin* along the dental epithelium. Therefore, it can be suggested that *miRNA-221-3p* plays a modulating role in Wnt/β-catenin signaling during crosstalk between epithelium and mesenchyme, due to the optimal level of Wnt/β-catenin signaling being essential for the developmental progression of the tooth (Wang and Feng, 2017). Similarly, the developmental role of Bmp, a member of the TGF- β- superfamily, also plays a vital role during early and late tooth differentiation (Thesleff, 2003; Jussila and Thesleff, 2012; Wang et al., 2012). In our study, the inhibition of *miRNA-221-3p* resulted in increased Bmp signaling and intense mesenchymal localization of pSMAD1/5/8 in the inhibitor-treated specimen. This altered Bmp signaling resulted in decreased cellular proliferation and apoptosis in the dental epithelium and adjacent mesenchyme, as in previous reports (Wang et al., 2012; Kim et al., 2014). This would result in a smaller tooth size at E13 + 2 days (Figure 1). A miRNA target prediction tool miRDB (Chen and Wang, 2020) predicts more than 530 targets of *miRNA-221-3p*. Among some of the notable targets are cyclin-dependent kinase inhibitors, Bcl2 like 11, Bmpr1a, and Lrp10 suggesting that change in tooth size resulted from change in combination of different signaling pathways related to cell cycle arrest, apoptosis, BMP and WNT signaling. Future works are needed for the validation of putative role of *miRNA 221-3p* on modulation of these molecules.

Inhibition of *miRNA-221-3p* decreased the expression of *Fgf4*, which in general, promotes proliferation, determines tooth cusps (Jernvall et al., 1994; Du et al., 2017), and prevents cell apoptosis in the dental epithelium and mesenchyme (Vaahtokari et al., 1996; Jernvall et al., 1998). Kratochwil et al., 2002 showed that *Fgf4* is completely lost in *Lef1* knockout teeth which is arrested at bud stage and recombinant *Fgf4* rescued this phenotype. Consistent with this, we expected smaller tooth size with decreased level of *Fgf4* in inhibitor treated specimen. Pharmacological inhibition of Fgf signaling by SU5402 blocked the epithelial morphogenesis and tooth mineralization in zebrafish (Jackman et al., 2004) which also suggest that *Fgf4* is important signaling for proper tooth crown morphogenesis. It also contributed to decreased tooth size through decreased cell proliferation and increased apoptosis. At the same time, *Shh* induced proliferation in a differential manner and determines tooth shape and affects ameloblast polarization through odontoblast differentiation and loss of *Shh* did not change the expression of other signaling molecules like *Fgf4*, *Bmp2*, and *Wnt10b* (Dassule et al., 2000). However, loss of *Shh* altered the expression patterns in developing dental tissues suggesting that the loss of *Shh* results in a change of cell fate, from proliferative to a less proliferative enamel knot-like population (Dassule et al., 2000) which reinforce our hypothesis that miRNA modulation of *Shh* signaling is crucial for crown morphogenesis and evident in inhibitor-treated specimen. Consistent with our results, inhibition of *Shh* by 5E1 and Forskolin during tooth organ culture resulted decreased proliferation, increased apoptosis leading to altered tooth phenotype (Cobourne et al., 2001). In quiescent state, *Ptch1* suppress the Smoothened which in turn controls the Gli transcriptional effectors in the repressive forms. In the presence of SHH ligand, the *Ptch1* suppression of Smoothened is relieved and the signal is transduced through activation of Gli transcription factors (Cohen et al., 2015) which is tightly regulated process. When tooth are cultivated in presence of *miRNA-221-3p* inhibitor, *Ptch1* is elevated which may interfere the regulation of *Shh* signaling through extended Smoothened suppression. On the other hand, mimicking the *miRNA-221-3p* lowers the *Ptch1* in the system and Smoothened may no longer be suppressed which may bypass the early step of *Ptch1*-*Shh* binding which ultimately affects the *Shh* signaling cascade resulting altered *Shh* expression in the cultured tissues. Also, these altered expression area would be explained by spatiotemporal specific expression of genes which are required for harmonious organogenesis rather than just changing in expression levels during development. These results demonstrate the tight regulation of signaling molecules during the early stages of tooth development, which lay the foundation for the complete morphogenesis of crown and root. In addition, mesenchymal expressed Bmp signaling is also important for HERS development and tooth root formation (Hosoya et al., 2008), further suggesting that the signaling modulation by *miRNA-221-3p* will guide the tooth root formation possibly through modulation of Wnt/β-catenin and Bmp signaling. Previous studies have demonstrated the role of ROCK1 in ameloblast differentiation (Otsu et al., 2011) and the association of ROCK1 and cadherin complex for maintaining cellular adhesion (Smith et al., 2012). However, E-cadherin-mediated cellular adhesion was decreased with inhibition and mimicking of *miRNA-221-3p* in our study (Figure 2), suggesting that *miRNA-221-3p* modulates ROCK1 and E-cadherin-mediated maintenance of actin cytoskeleton and cellular adhesion that ultimately regulates the differentiation stage of tooth and crown morphogenesis.

During mice molar development, at around PN2, crown formation is almost completed and the root formation initiates with the emergence of HERS (Lungová et al., 2011). The corono-apical extension of the OEE and IEE along the cervical loop forms HERS and guides for the development of roots (Lungová et al., 2011; Li et al., 2017). HERS, which is regarded as the signaling center for tooth root formation and contains various signaling molecules and transcription factors (Huang et al., 2009; Lungová et al., 2011; Li et al., 2017). For tooth root progression, the cellular proliferation of HERS together with adjacent mesenchymal cells is evident (Lungová et al., 2011; Sohn et al., 2014; Li et al., 2017). In this study, the inhibition of *miRNA-221-3p* showed increased proliferative cells along the HERS and its adjacent mesenchymal cells (Figure 4). The epithelial cells of HERS had *Shh* expression, and the reciprocal interactions of *Shh* signaling along the HERS epithelium and adjacent mesenchymal cells, would play a significant role in tooth root formation (Nakatomi et al., 2006; Ota, 2008; Li et al., 2017). However, this study demonstrated an increased expression of *Shh* along the HERS in the inhibitor group compared to control and mimic (Supplementary Figure 6). This may have contributed to increased proliferation of HERS and ultimately affected tooth height, which is consistent with previous studies that showed the partial rescue of root development by *Shh* in the rootless K14-Cre;Smad4^fl/fl^ (Huang et al., 2010; Hosoya et al., 2020). In addition, cell proliferation is reduced around the HERS in homozygous Ptc^mes^ mutants affecting the tooth root formation (Nakatomi et al., 2006) further reiterating that the long and slender teeth in 3-week renal calcified *miRNA-221-3p* inhibitor-treated teeth are a result of increased proliferation and intense *Shh* expression. The inhibition of *miRNA-221-3p* at the bud and cap stage resulted in a decreased expression of *Shh* after 2 days (Figure 2), however, the knockdown effect at this stage would result in a return of its expression patterns after the inhibitor was removed. Therefore, it can be speculated that 1-week renal calcified teeth had comparatively increased expression patterns of *Shh* along the HERS in the inhibitor group compared to control and mimic (Supplementary Figure 6). As the transient knockdown of genes can result in a regain of its expression at a certain time point (Eadon et al., 2017), it can also be deduced that the inhibition of *miRNA-221-3p* may also be regained and upregulate the *Shh* expression in the HERS after 1-week.

In conclusion, *miRNA-221-3p* modulates the Shh and Wnt signaling during cap and bell stages of tooth development, and the reciprocal interaction between the mesenchyme and epithelium, in turn, regulates other signaling molecules, such as Fgf and Bmp. These changes in signaling eventually determine the fate of tooth crown and root morphogenesis through hard tissue formation. Therefore, we conclude that the modulation of *miRNA-221-3p* signaling may explain some of the processes involved in the integrative and fine-tuning networks of signaling molecules involved in tooth development. In addition, it is required to examine the detailed signaling regulations of SHH signaling through *miRNA-221-3p* using pharmacological modulators of miRNA inhibited tooth culture and, rescue of β-catenin and Fgf signaling. Furthermore it also would be necessary to reveal the consequence signaling including p-ERK, Etv4, Etv5, and so on using genome wide screening methods to define the detailed signaling modulations in tooth morphogenesis.

## Data Availability Statement

The raw data supporting the conclusions of this article will be made available by the authors, without undue reservation.

## Ethics Statement

The animal study was reviewed and approved by KNU-2020-0107.

## Author Contributions

YA, SN, and J-YK contributed to conception, design, and data interpretation, critically revised the manuscript. T-YK, E-SL, C-HA, J-YK, HY, SL, YL, and W-JS contributed to data analysis, interpretation, and critically revised the manuscript. All authors gave final approval and agreed to be accountable for all aspects of the work.

## Conflict of Interest

The authors declare that the research was conducted in the absence of any commercial or financial relationships that could be construed as a potential conflict of interest.

## Publisher’s Note

All claims expressed in this article are solely those of the authors and do not necessarily represent those of their affiliated organizations, or those of the publisher, the editors and the reviewers. Any product that may be evaluated in this article, or claim that may be made by its manufacturer, is not guaranteed or endorsed by the publisher.

## Footnotes

1
http://imagej.net/
