# Supplementary material for: Signaling Modulation by miRNA-221-3p During Tooth Morphogenesis in Mice
Source: Front Cell Dev Biol. 2021 Aug 25;9:697243. doi: 10.3389/fcell.2021.697243 (PMC8424101; doi:10.3389/fcell.2021.697243)
Supplement: Supplementary file 1 [file Data_Sheet_1.docx]

**Supplementary figures**

**Fig. S1. ImajeJ processing of image for nucleus and Ki67 positive cells counting. Results are expressed as % Ki67 positive cells in a given field.**

**
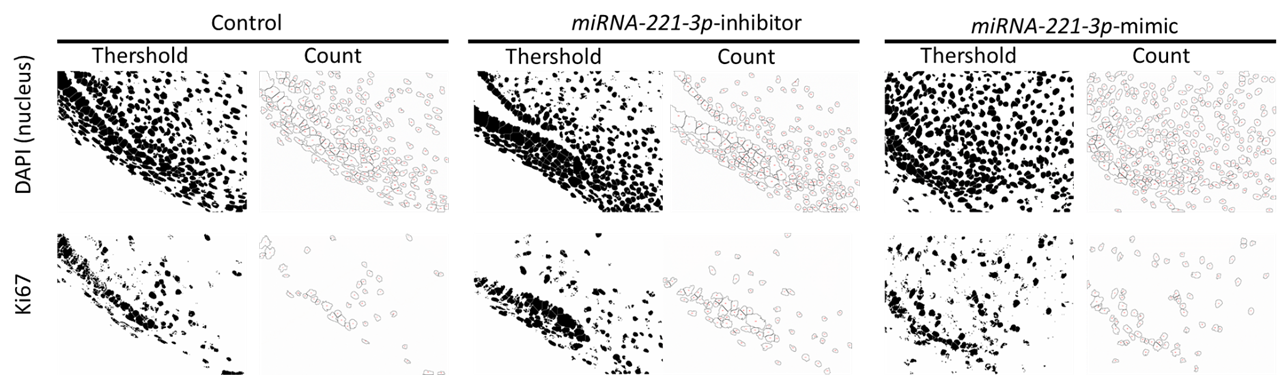
**

**Fig. S2. Seed sequence of *miRNA-221-3p* and *Ptch1***

**
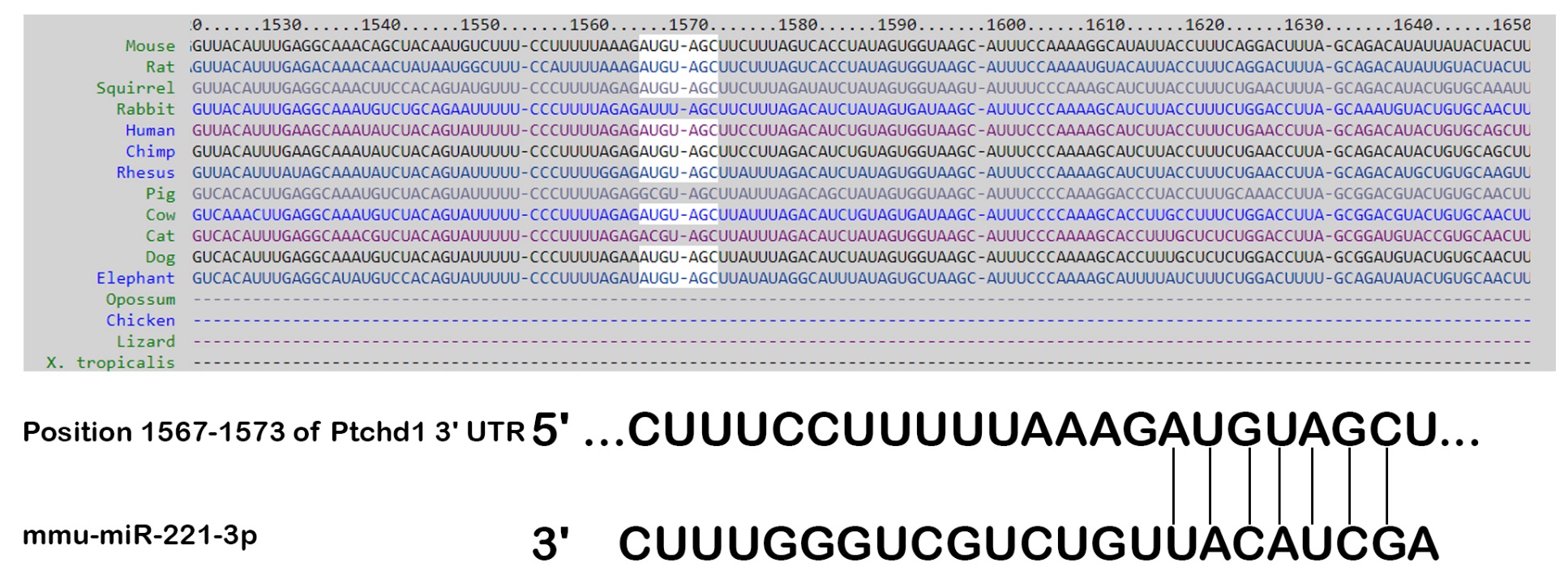
**

**Fig. S3.** Schematic diagrams showing mesio-distal and region of interest for TUNEL and Ki67 positive cell counts.

**
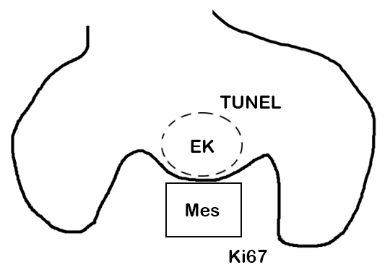
**

**Figure S4:** RT-qPCR showing expression of signaling molecules after *miRNA-221-3p* inhibition (A) and mimic (B) at E13 for 36 hours


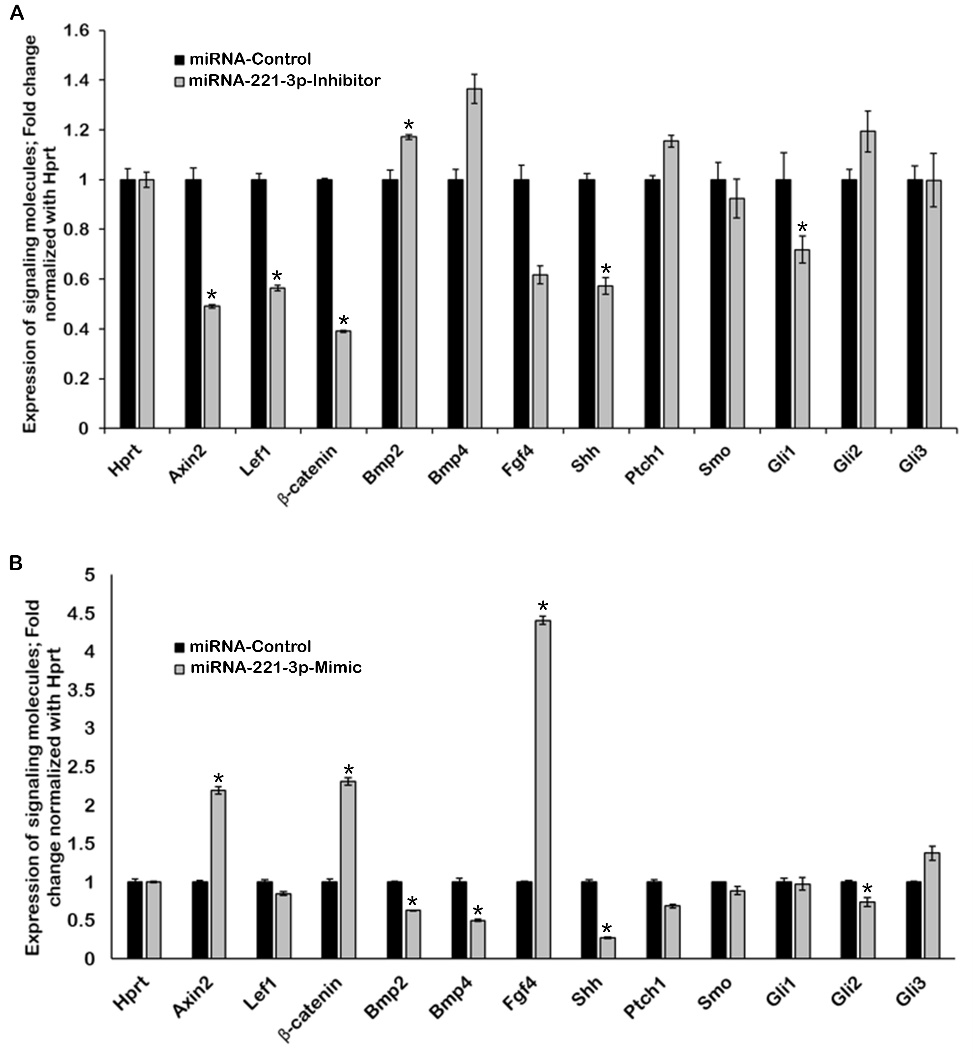

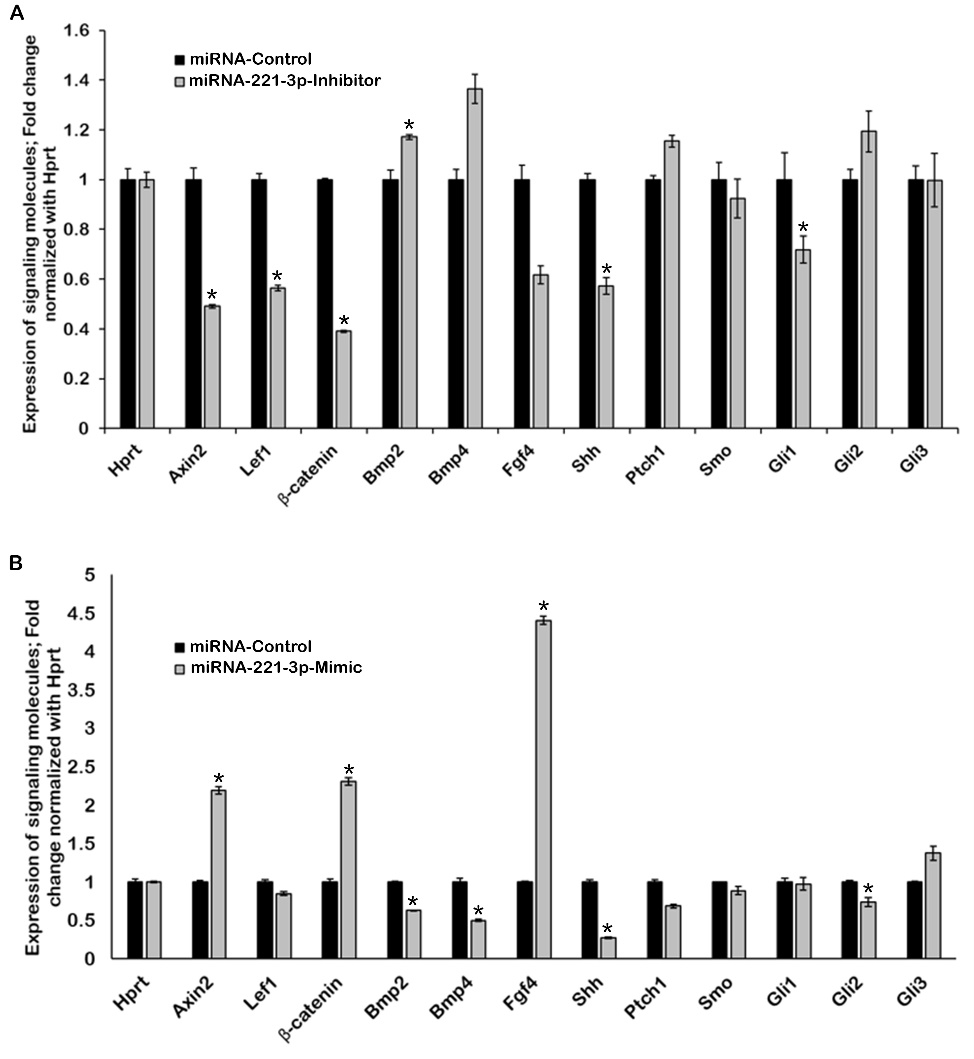


**Fig. S5.** Calculation of nuclear localization of Beta-catenin using Blue (DAPI) and Green (Beta-catenin) colocalization correlation index using Fiji plugin “Colocalization” (A) and digitally enlarged Beta-catenin immunohistochemistry (B).


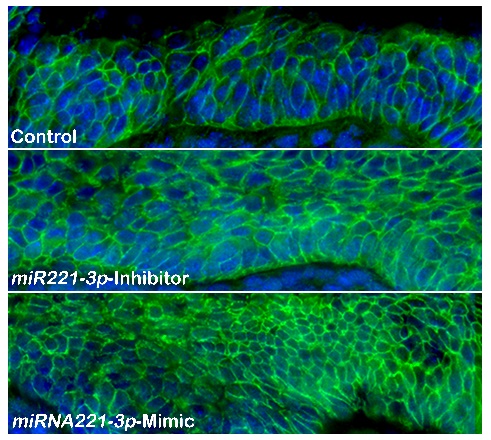

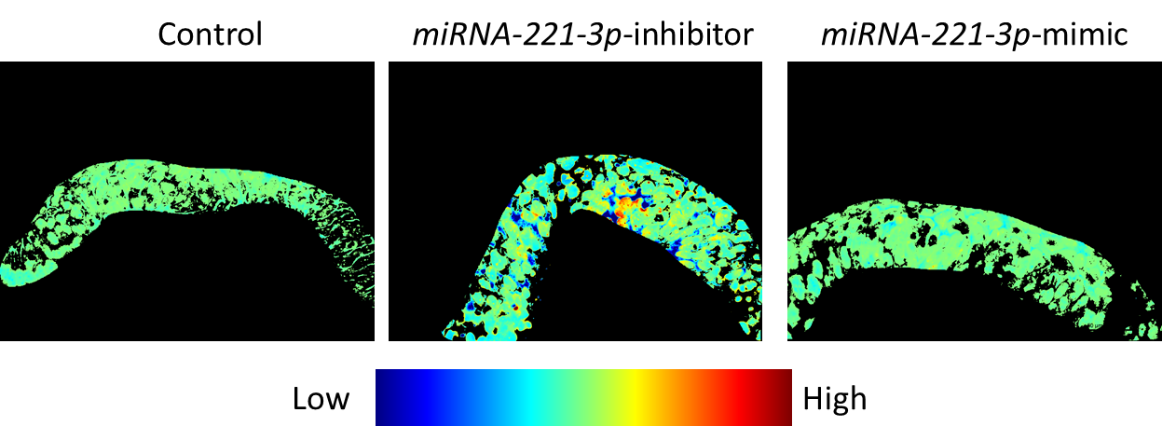


B

A

**Fig. S6.** Altered expression pattern of *Shh* along the HERS. Section in situ hybridization showing expression of *Shh* along the HERS (A-C). The expression of *Shh* is much stronger in the inhibitor treated specimen (B) compared to mimic and control (A, C) (N=3).


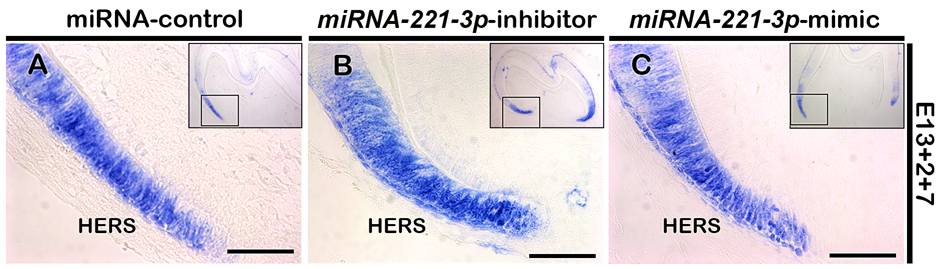


**Supplementary table 1.** Primer sequences for qPCR

Primer sequences for miRNA qPCR

**Supplementary table 2**. qRT-PCR Raw data for *miRNA 221-3p* (A); and tooth development related signaling molecules after 48 hours (B) and 36 hours (C) from control, inhibitor and mimic treated specimens.

A.

B.

C.
